# Bacteria related to *Bradyrhizobium yuanmingense* from Ghana are effective groundnut micro-symbionts

**DOI:** 10.1016/j.apsoil.2018.03.003

**Published:** 2018-06

**Authors:** Ophelia Osei, Robert C. Abaidoo, Benjamin D.K. Ahiabor, Robert M. Boddey, Luc F.M. Rouws

**Affiliations:** a Department of Crop and Soil Sciences, Faculty of Agriculture, Kwame Nkrumah University of Science and Technology, PMB, Kumasi, Ghana; b Department of Theoretical and Applied Biology, Kwame Nkrumah University of Science and Technology, PMB, Kumasi, Ghana; c International Institute of Tropical Agriculture, PMB 5320, Ibadan, Nigeria; d CSIR, Savannah Agricultural Research Institute, P.O. Box 52, Tamale, Ghana; e Embrapa Agrobiologia, Rodovia BR 465 km 07, Seropédica, Rio de Janeiro 23891-000, Brazil

**Keywords:** Native isolates, Biological nitrogen fixation, Genetic diversity, *Arachis hypogaea* L.

## Abstract

The identification of locally-adapted rhizobia for effective inoculation of grain legumes in Africa’s semiarid regions is strategic for developing and optimizing cheap nitrogen fixation technologies for smallholder farmers. This study was aimed at selecting and characterising effective native rhizobia, from Ghanaian soils for groundnut (*Arachis hypogaea* L.) inoculation. From surface-disinfected root nodules of cowpea and groundnut plants grown on farmers’ fields, 150 bacterial isolates were obtained, 30 of which were eventually found to nodulate groundnut plants. After testing the symbiotic potential of these isolates on groundnut on sterilized substrate, seven of them, designated as KNUST 1001–1007, were evaluated in an open field pot experiment using ^15^N-labelled soil. Although ^15^N dilution analyses did not indicate differences among treatments in the proportion of nitrogen (N) derived from the atmosphere (%Ndfa), all seven strains increased total N derived from N_2_ fixation by inoculated groundnut plants as compared to the non-inoculated control. Inoculation with KNUST 1002 led to total N accumulation as high as that of the groundnut reference strain 32H1. Genetic characterisation of the isolates by sequence analysis of 16S rRNA gene, 16S – 23S rRNA intergenic transcribed spacer (ITS) region and *nodC* gene revealed that isolates KNUST 1003 and 1007 were related to *Rhizobium tropici*, a common bean symbiont. The other five isolates, including KNUST 1002 belonged to the *Bradyrhizobium* genus, being closely related to *Bradyrhizobium yuanmingense*. Therefore, this study revealed novel native Ghanaian rhizobia with potential for the development of groundnut inoculants.

## Highlights

• Native rhizobium strains effectively nodulated a popular groundnut variety in Ghana.
• Rhizobium inoculation increased biological N_2_ fixation in groundnut grown in soil.
• Ghanaian groundnut strains are genetically related to *Bradyrhizobium yuanmingense.*
• Native isolates are a potential source of strains for local inoculant production.

## 1 Introduction

Groundnut (*Arachis hypogaea* L.) is a multipurpose grain legume, which is considered a nutritious component in diets and a source of income for smallholder farmers in developing countries (Carlberg, 2012). In Ghana, about 94% of the groundnut production is concentrated in the northern region; a place considered as one of West Africa’s main groundnut production areas (Tsigbey et al., 2003). In terms of symbiotic nitrogen fixation, groundnut has been found to form effective association with both fast and slow growing ‘rhizobia’ of the *Rhizobium* and *Bradyrhizobium* genera, respectively (Taurian et al., 2002). Among the *Bradyrhizobium* strains identified to nodulate groundnut are: *Bradyrhizobium arachidis, Bradyrhizobium japonicum, Bradyrhizobium elkanii, Bradyrhizobium lablabi, Bradyrhizobium yuanmingense and Bradyrhizobium iriomotense* (Taurian et al., 2006, El-Akhal et al., 2008, Chang et al., 2011, Muñoz et al., 2011, Wang et al., 2013). Other species that nodulate groundnut include *Rhizobium gardinii* and *Rhizobium tropici* (Taurian et al., 2006). Despite the nitrogen-fixing ability of groundnut, yields are often below their maximum potential (Nutsugah et al., 2007). These low yields have been partially attributed to low inherent soil fertility and nutrient deficiencies in N and P mostly limit productivity of this crop (Maheswar and Sathiyavani, 2012, Mohamed and Abdalla, 2013).

Options such as mineral nitrogen application and rhizobium inoculation have been considered as means to supply legumes with N (Mweetwa et al., 2014). Apart from the possible adverse environmental consequences of excessive mineral nitrogen application (Trindade et al., 2001, Flechard et al., 2007), farmers are unable to exploit this option due to financial constraints. Thus, the more feasible alternative is the use of rhizobium inoculants. The practice of inoculation with highly effective rhizobium strains has been identified, among other factors, as an essential means to promote biological nitrogen fixation (BNF) with subsequent increases in grain yields (Unkovich and Pate, 2000). The usefulness of this BNF process is made evident when legumes depending on atmospheric N_2_ produce increased yields in soils in which non-legume crops would require a substantial amount of mineral nitrogen. In addition, inoculation of groundnut has led to considerable increases in nodulation, growth and productivity (Sajid et al., 2010, Sharma et al., 2011, Mohamed and Abdalla, 2013). Despite the potential benefits of inoculation, farmers rarely apply inoculants to groundnut because of the consensus that the association between groundnut and native soil rhizobia is usually adequate. Another factor that could contribute to the limited use of inoculants by farmers is the low awareness of the higher economic returns from the use of inoculants relative to mineral nitrogen (Ndakidemi et al., 2006). Limited availability of groundnut inoculants (i.e. of exotic origin) and lack of local strains for inoculating the crop particularly in Ghana, further exacerbate the limited use of inoculants.

To improve inoculation response of tropical legumes, Nkot et al. (2008) suggested the use of indigenous rhizobia as inoculants. For example, improvement in nodulation and N_2_ fixation was reported when groundnut was inoculated with native rhizobia (Bogino et al., 2008). Shishido and Pepper, 1990, Sattar et al., 1995 suggested that strains isolated from a particular region are the most effective for a given crop in that same region. In addition, rhizobia that are moderately to highly effective have been found to be well represented among the native population (Herridge et al., 2008) and could serve as a source of elite strains for local inoculant production. This emphasizes the need to identify elite isolates adapted to the prevailing environmental conditions for improved BNF. In selecting rhizobia strains for use as inoculants, the characteristics competitiveness in nodule formation and effectiveness in nitrogen fixation are considered (Stephens and Rask, 2000).

Conversely, the symbiotic potential and genetic diversity of groundnut-nodulating rhizobia is yet to be investigated, particularly in the context of Ghanaian agriculture. Previous reports based on the analyses of 16S rRNA and RFLP revealed a large diversity within cowpea- and soybean-nodulating strains only at the genus level (Abaidoo et al., 2000, Fening et al., 2004). Therefore, it is imperative to assess the diversity within the native rhizobium populations that nodulate groundnut and to estimate their contribution to N_2_ fixation in grain legumes. The aim of this study was to characterise rhizobia capable of nodulating groundnut using molecular tools and to identify elite strains for groundnut inoculation. To this end, symbiotic potential and phenotypic tests in addition to sequence analyses of 16S rRNA gene, 16S – 23S rRNA intergenic transcribed spacer (ITS) region and symbiotic genes; *nodC* and *nifH* were carried out to reveal the diversity within groundnut nodulating rhizobium and identify elite strains for improved inoculation response.

## 2 Materials and methods

### 2.1 Recovery and authentication of Rhizobium isolates

Groundnut and cowpea nodules were collected from farmers’ fields across the three regions in northern Ghana at the flowering stage and sampling points were located using a GPS (Supplementary Fig. S1). Recovered nodules were kept on desiccated silica gel and transported to the microbiology laboratory, Kwame Nkrumah University of Science and technology (KNUST) in Kumasi, Ghana, for isolation. Dried nodules were rehydrated in sterile distilled water overnight. After rehydration, whole nodules were surface sterilised using 95% ethanol for 10 s and transferred into a 3% hydrogen peroxide solution for 3 min. The nodules were then rinsed in several changes of sterilised distilled water to remove the remaining hydrogen peroxide as described by Somasegaran and Hoben (1994). Sterilised nodules were carefully crushed onto YMA (yeast mannitol agar) plates (Fred and Waksman, 1928) under aseptic conditions using heat-sterilised forceps. The resulting plates were incubated at 28 °C and monitored for 10 days. Bacterial colonies were repeatedly streaked on YMA medium to obtain pure cultures.

To authenticate isolates as true rhizobia, a nodulation test was carried out under aseptic and controlled conditions using cowpea (*Vigna unguiculata* L. Walp, cv. Asontem) as the test host. Cowpea was selected for this initial screening because of its highly promiscuous nodulation pattern and for being easily cultivable in growth pouches. Cowpea seeds were prepared (Section 2.4) and pre-germinated on moist sterile tissue paper in Petri dishes and incubated at 28 °C for three days. Seedlings with equal radicle length (2 cm) were selected and aseptically transferred into plastic growth pouches (Mega International, USA) containing N-free plant nutrient solution (Broughton and Dilworth, 1970). After seeding, the growth pouches were arranged on a wooden rack and placed in the greenhouse at KNUST, Kumasi, Ghana. A week after transplanting, broth cultures of each of the isolates were used to inoculate the cowpea seedlings. At 28 days after inoculation, the seedlings were assessed for nodulation and isolates that induced nodule formation on the test host were considered as true rhizobia. Where no nodules were observed, the isolate was not subjected to further studies. Isolates confirmed as true rhizobia were maintained on agar slants and also in 25% (w/v) glycerol (at −20 °C) for short term and long term (−80 °C) storages, respectively.

### 2.2 Symbiotic potential of native isolates on groundnut in sterilised sand in Ghana

The sixty-five isolates, that were considered true rhizobia based on the authentication test on cowpea, were evaluated for their symbiotic potential together with recommended/commercial strains namely: *Bradyrhizobium diazoefficiens* USDA 110 (soybean strain from Florida, USA) (Delamuta et al., 2013), and two Brazilian elite-strains, *Bradyrhizobium pachyrhizi* strain BR 3262 and *Bradyrhizobium yuanmingense* strain BR 3267 (Leite et al., 2017). USDA 110 is a strain widely used in commercial inoculants for soybean in Africa and in characterising newly cultured isolates.

The groundnut variety ‘Chinese’ (an early maturing variety preferred by most farmers in Ghana) was used. For the first experiment, four-litre capacity pots were filled with 3 kg of sterilised river sand and arranged in the greenhouse at KNUST, Kumasi, Ghana. Prior to filling the pots, the sand was sterilised in an autoclave at 121 °C for 1 h (Lupwayi and Haque, 1994). Broughton and Dilworth (Broughton and Dilworth, 1970) N-free nutrient solution was used to irrigate the plants weekly. The strains were classified by a symbiotic effectiveness index (SEI) that was calculated from the shoot dry matter (SDM) of the groundnut plants inoculated with a specific isolate divided by the SDM of groundnut plants inoculated with the reference strain BR 3267, expressed as a percentage (Yates et al., 2016).

### 2.3 Nitrogen fixation contribution of isolates on groundnut in ^15^N labelled soil in Brazil

The second experiment was conducted in pots in the open field at Embrapa Agrobiologia, Seropédica, Brazil. The planting medium used was soil classified as an Alfisol (US Soil Taxonomy Classification) obtained from Piracicaba, São Paulo State, Brazil, with a history of ^15^N enrichment since the 1980s through the application of ^15^N labelled organic matter (Tsai, Siu Mui, CENA, Piraicicaba, personal communication). Due to the compacted nature of the soil, it was mixed with 50% sand to improve drainage. Prior to the experiment, the chemical properties of the soil were analysed using the methods of Souza and Nogueira (2005): pH in H_2_O, 5.3; exchangeable Al, Ca and Mg: 0.04, 0.96 and 0.18 cmolcd^−1^, respectively; and P and K were 16.2 and 21.7 mgL^−1^, respectively. The soil had sand, silt and clay fractions of 14%, 22% and 64% respectively and fell within the clay textural class. Seven effective isolates identified from the first experiment alongside the reference strains BR 3267 and three other effective/recommended groundnut strains, *Bradyrhizobium* sp. strain BR 10254 (Torres-J et al., 2014), *Bradyrhizobium* sp. strain 32H1 (Urtz and Elkan, 1996) and SEMIA 6144 (Menna et al., 2009) were used as the treatments. Also included were three non-N_2_-fixing reference plants: non-nodulating (NN) soybean (*Glycine* max), NN common bean (*Phaseolus vulgaris*) and sorghum (*Sorghum bicolor,* cv. BR 305). Each experimental unit consisted of five-litre capacity pot filled with 4 kg of soil, amended with 732 mg P_2_O_5_, 241 mg K_2_O and the specific treatment. Clean tap water was used to irrigate the experiment every week. In both experiments, two un-inoculated controls; (1) with nitrogen (70 ppm in the form of 0.05% KNO_3_ for the first experiment and 100 mg N in the form of NH_4_NO_3_ for the second experiment) and (2) without nitrogen (-N) were included.

### 2.4 Bacterial culture, experiment management and experimental design

Broth cultures of each of the isolates and reference strains used in this study were prepared by inoculating a loop-full of pure culture in yeast mannitol broth (YMB). The cultures were then incubated in an orbital incubator at 125 rpm and 28 °C until the late logarithmic growth phrase where an O.D._600 nm_ of 1.0 was achieved. Groundnut seeds were surfaced sterilised with 95% ethanol for 30 s and 3% hydrogen peroxide solution for 3 min followed by several rinses in sterilised distilled water (Somasegaran and Hoben, 1994). Five seeds were planted per pot with the help of a pair of sterile forceps and thinned to two plants one week after planting. One mL culture of each of the test isolates or reference strain was used to inoculate the seeds at planting. Unless otherwise stated, all bacteria cultures, seed preparation and planting in this study followed the procedure outlined in this section. Both trials were arranged in a randomized complete block design (RCBD) with four replicates.

### 2.5 Data collection

Plants were harvested 45 days after planting for the experiments conducted in sand and soil/sand mixture (i.e. in Ghana and Brazil, respectively). Groundnut shoots were separated from the roots at the soil surface level. Nodulated roots and detached nodules were collected and stored in polythene bags. Samples were transported to the microbiology laboratory, KNUST in Ghana and Embrapa-Agrobiologia in Brazil, respectively, for processing. Nodules were separated from roots by gently washing the root system under running tap water to remove all debris and adhering sand or soil after which the nodules were detached, counted and oven dried together with shoots at 65 °C for 72 h to estimate dry biomass.

For the second experiment, dried plant shoots were ground to fine powder using a roller miller similar to that described by Arnold and Schepers (2004). Total N contents of all plant samples and seeds were analysed using the semi-micro Kjeldahl procedure as described by Urquiaga et al. (1992). The ^15^N enrichment of aliquots of sub-samples containing between 35 and 70 µg of N were weighed into tin capsules and determined using an automated continuous-flow isotope-ratio mass spectrometer consisting of a Costech EA Model ECS 4010 automatic C and N analyzer coupled to a Thermo Delta V Advantage mass spectrometer (Costech Analytical, Valencia, CA, USA). Since the ^15^N dilution technique was used for this study, the ^15^N enrichment of the N derived from the soil was estimated by discounting the N derived from seed and its excess ^15^N content using the formula proposed by Boddey et al. (1995):

$${}^{15}\text{N enrichment}(\text{SC})=\{(\text{plant N}\;*\;\text{at}.\%\text{xs plant})-(\text{seed N}*\;\text{Ps}*\;\%\text{xs seed)\}}/(\text{plant N}-\text{seed N})$$

where SC indicates corrected for seed N, at.% xs is the atom% ^15^N excess and Ps is the proportion of the seed N that was assimilated by the plant tissue. Ps was assumed to be 50% since the correction was done for shoot tissue only (Okito et al., 2004).

For the proportion of N derived from the air (%Ndfa) via BNF, the equation of (Chalk, 1985) was used:

$$\%\text{Ndfa}=100\;*\;\{1-(\text{at}.{\%}^{15}\text{Nxs of legume}/\text{at}.{\%}^{15}\text{Nxs of reference})\}$$

where at.% ^15^Nxs is the atom% ^15^N excess of the shoot tissue of the plants. As three different non-N_2_-fixing reference crops were included, individual as well as combined estimates of the %Ndfa for each isolate could be calculated using both the total N difference method and ^15^N isotope dilution method.

### 2.6 Statistical analysis

Data measured for the various experiments were subjected to analyses of variance using SISVAR (Ferreira, 2008). Where overall probability was significant (p < 0.05), means were separated using Scott Knott at 5% probability. Relationships among isolates and sampling sites were explored with principal component analysis using the software STATGRAPHICS Centurion V16.1.11 (StatPoint Technologies Inc, Warrenton, VA)

### 2.7 Morpho - cultural characteristics of effective isolates

Characterisation of effective isolates was carried out when at least three isolated colonies were observed after streaking on YMA with bromothymol blue as pH indicator. Characteristics analysed were: pH reaction of culture medium, number of days to form colonies, colony elevation and form and mucus production. Bacterial cultures were given identification numbers (Table 1) and deposited in the culture collections of Johanna Döbereiner Biological Resources Center (CRB-JD), Embrapa Agrobiologia, Brazil and the KNUST Microbiology Laboratory, Kumasi, Ghana.

**Table 1:** GenBank accession numbers of sequences obtained in this study.

*Bradyrhizobium* isolates^a^		Genbank accession number			
KNUST ID	CRB-JD ID	16S rRNA	ITS	*nod*C	*nif*H
KNUST 1001	BR 10839	KY229769	MF108830	KY040459	KY040464
KNUST 1002	BR 10840	KY229770	MF108831	KY040460	KY040465
KNUST 1004	BR 10824	KY229772	MF108832	KY040461	KY040466
KNUST 1005	BR 10841	KY229773	MF108833	KY040462	KY040467
KNUST 1006	BR 10842	KY229774	MF108834	KY040463	KY040468

*Rhizobium* isolates					
KNUST 1003	BR 10837	KY229771	–	–	–
KNUST 1007	BR 10838	KY229775	–	–	–

a KNUST ID: Kwame Nkrumah University Technology culture collection identification, CRB-JD: Johanna Döbereiner Biological Resource Center culture collection identification.

### 2.8 DNA extraction, PCR amplification and gene sequencing

Bacterial genomic DNA was extracted using the Wizard genomic DNA purification kit (Promega, USA). Extracted DNA was submitted to amplification of 16S rRNA gene (Lane, 1991) and the intergenic transcribed spacer (ITS) region between the 16S and 23S rRNA genes (Cardinale et al., 2004). Additionally, the symbiotic gene *nodC* (Sarita et al., 2005) and the nitrogenase reductase gene *nifH* (Poly et al., 2001) were amplified. For each of the genes amplified, conditions for the PCR specified by the cited references were employed. PCR amplicons were subjected to bi-directional sequencing using the BigDye® Terminator v3.1 Cycle Sequencing Kit (ThermoFisher). Sequencing reaction products were subjected to post-reaction clean-up and analysed using an ABI 3500 Genetic Analyzer (Thermo Fisher). Quality control and sequence assembly were performed using BioNumerics 7 (Applied Maths, Belgium).

### 2.9 Phylogenetic analyses

Multiple nucleotide sequence alignments were generated using CLUSTAL W (Thompson et al., 1994) and phylogenetically analysed using MEGA7 software (Kumar et al., 2016). Concatenated sequence analyses of the 16S rRNA and the ITS region for the isolates in this study as well as type-strains of recognized *Bradyrhizobium* species were aligned and trimmed to the same length. Aligned sequences of the different genes were then concatenated using the Seaview program (Galtier et al., 1996). The maximum likelihood reconstruction method was used in calculating the phylogenetic trees of individual and concatenated genes. The most suitable models for generating phylogenetic trees of individual and concatenated genes were determined for each alignment using the integrated model selection tool of MEGA7. The strength of the phylogenetic tree topologies was evaluated using the bootstrap method by applying 500 pseudo replicates (Felsenstein, 1985). DNA sequences obtained for the various isolates after sequencing have been deposited in the GenBank database under the accession numbers in Table 1.

## 3 Results

### 3.1 Symbiotic potential of Ghanaian rhizobium isolates on groundnut in sterilised sand

A total of 65 bacterial isolates were authenticated after inducing root nodules when inoculated individually on cowpea plants grown in sterile growth pouches in a greenhouse.

These 65 authenticated strains were then tested for their performance by inoculating them on groundnut grown in pots with sterilised sand medium. The negative control (groundnut plants without inoculation or nitrogen fertilizer) did not form any nodules and showed nitrogen deficiency symptoms. Among the 65 authenticated rhizobia, only 30 induced nodulation in groundnut, demonstrating a difference in micro-symbiont specificity between groundnut and cowpea. Significant treatment effects on nodule number were observed following analysis of variance with three of the isolates producing statistically more (p < 0.05) nodules than all the reference strains (Table 2). Isolate KNUST 1006 produced nodule numbers similar to that of the reference strain BR 3267. With the exception of KNUST 1002, all the isolates that induced increased nodule numbers also resulted in increased nodule dry weights that were significantly (p < 0.05) higher than that observed for the reference strain, BR 3267. Isolate KNUST 1007 also caused a significant increase in nodule dry weight. Treatment with isolates KNUST 1001 and 1002 caused the greatest increase (p < 0.05) in shoot dry weight (Table 2). Symbiotic effectiveness of isolates also varied significantly (p < 0.05) among the isolates with isolates KNUST 1001 and 1002 performing better than the reference strain BR 3267 (Table 2). Twelve of the isolates had significantly lower symbiotic effectiveness indices (SEI) than all the reference strains. The lowest SEI was recorded for the control treatment without nitrogen and isolate KNUST 1020. Profiling the symbiotic effectiveness of isolates placed 23% into the effective group (i.e. SEI > 75%). The remaining isolates were considered as partially effective (Table 2).

**Table 2:** Nodulation and shoot dry weight of inoculated groundnut and symbiotic effectiveness of isolates in sterilised sand.

Isolate/strain		Source	Nodule number	Nodule dry weight (mg pot^−1^)	Shoot dry weight (g pot^−1^)	Symbiotic effectiveness index (%)
FIELD ID	KNUST ID					
2NAG 52b1	KNUST 1001†	Konta	137.3 a	150.0c	7.04 a	132.95 a
2NAG 53e	KNUST 1002†	Yipaani	105.0 b	100.0 d	7.20 a	136.00 a
2NAG 9d	KNUST 1003†	Punyoro kb	99.3 b	290.3 a	4.94 c	93.34 c
2NAG 8a	KNUST 1004†	Kandiga 2	24.0 e	70.0 e	3.89 e	73.56 e
2NAG 75b	KNUST 1005†	Akuokayili	18.0 e	44.0 f	4.23 d	79.98 d
2NAG 08e	KNUST 1006†	Kandiga 2	84.7c	183.3 b	5.84 b	110.48 b
2NAG 87c	KNUST 1007†	Boro	12.3 f	180.0 b	5.02 c	94.86 c
2NAG 01e	KNUST 1008	Tamale	37.3 d	47.7 f	2.34 h	44.23 h
2NAG 08d	KNUST 1009	Kandiga 2	10.3 f	45.7 f	3.25 f	61.35 f
2NAG 09b	KNUST 1010	Punyoro kb	5.0 f	24.7 f	2.54 h	47.95 h
2NAG 11d	KNUST 1011	Kandiga	6.3 f	34.0 f	2.93 g	55.33 g
2NAG 11f	KNUST 1012	Kandiga	13.3 f	23.0 f	2.39 h	45.19 h
2NAG 11 g	KNUST 1013	Kandiga	15.3 f	86.3 d	3.88 e	73.32 e
2NAG 13e	KNUST 1014	Naaga	7.0 f	24.7 f	2.41 h	45.62 h
2NAG 19d	KNUST 1015	Akuokayili 1	9.3 f	24.3 f	2.36 h	44.53 h
2NAG 20a	KNUST 1016	Pishigu	6.3 f	28.7 f	2.45 h	46.23 h
2NAG 70 g	KNUST 1017	Kuncheni	5.3 f	28.3 f	3.17 f	59.96 f
2NAG 71b	KNUST 1018	Zaguo deryiri	5.0 f	36.3 f	3.73 e	70.36 e
2NAG 72a	KNUST 1019	Zaguo deryiri	13.0 f	46.0 f	3.70 e	69.99 e
2NAG 73e	KNUST 1020	Gbare	6.7 f	44.0 f	2.01 i	37.91 i
2NAG 75b	KNUST 1021	Saawie	12.3 f	46.0 f	3.79 e	71.65 e
2NAG 80d	KNUST 1022	Varimpere	12.0 f	21.3 f	3.13 f	59.13 f
2NAG 81b	KNUST 1023	Varimpere	8.3 f	57.7 e	2.65 h	50.03 h
2NAG 84e	KNUST 1024	Chiatanga	8.7 f	19.7 f	2.48 h	46.86 h
2NAG 85c	KNUST 1025	Dorima	7.0 f	34.3 f	2.57 h	48.59 h
2NAG 87a	KNUST 1026	Boro	9.3 f	56.0 e	3.32 f	62.75 f
2NAG 87d	KNUST 1027	Boro	12.3 f	40.7 f	3.47 f	65.66 f
2NAG 92b	KNUST 1028	Tabiasi 1	5.3 f	19.0 f	2.94 g	55.49 g
2NAG 93e	KNUST 1029	Tabiasi 2	15.3 f	41.3 f	2.84 g	53.59 g
2NAG 97a	KNUST 1030	Kpalga	22.7 e	130.0 c	4.18 d	78.90 d
	Non-Inoculated		–	–	1.81 i	34.21 i
	Reference strains					
	USDA 110	USA	10.0 f	31.7 f	3.39 f	64.1 f
	BR 3262	Brazil	21.3 e	60.3 e	3.54 f	66.84 f
	BR 3267	Brazil	80.7 c	78.3 d	5.29 c	100.00 c
CV (%)			27.93	23.35	7.17	7.34

Means in the same column followed by the same letter are not significantly different at P < 0.05 (Scott Knott Test). 2NAG = Phase2N_2_Africa, ^†^ Isolates selected for second experiment.

Principal component analysis gave a better understanding of the symbiotic potential of the isolates and their biogeographic distribution across sampling sites. Two principal components explained 94.5% of the variation in symbiotic potential of test isolates and the three reference strains. The first principal component explained 84.1% of the variation, which was dominated by shoot dry weight (Supplementary Fig. S2). The second component explained 10.5% of the variation with nodule dry weight being the main contributing variable. Shoot dry weight and symbiotic effectiveness index pointed towards the same direction and were close to each other demonstrating a correlation between these two variables (Supplementary Table S1). Isolates that clustered in the direction of the shoot dry weight and symbiotic effectiveness variables, together with the reference strain BR 3267, recorded high values. On the other hand, isolates that clustered on the opposite side (i.e. to the left) produced lower values for the variables considered. A large proportion of the sampling sites harboured strains with SEI of between 25 and 75% and clustered on the left side.

### 3.2 Nitrogen fixation potential of selected isolates on groundnut grown in soil in Brazil

**Fig. 1:**
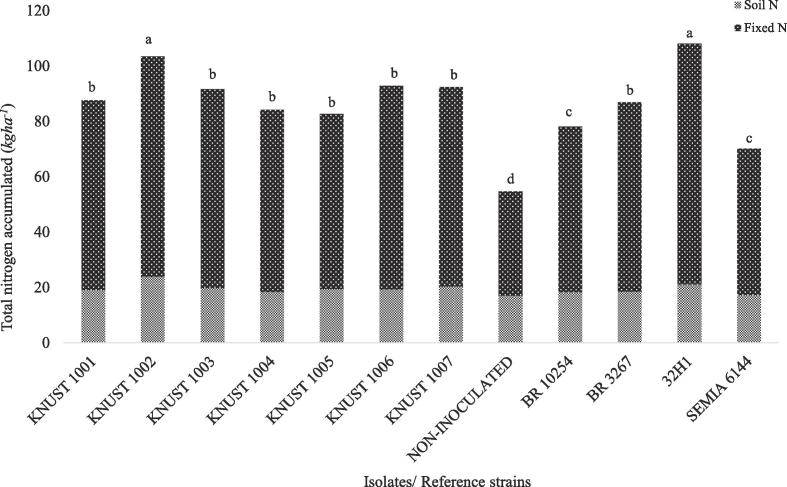
Total nitrogen accumulation and estimates of N derived from the air. Bars followed by the same letter are not significantly different at P < 0.05 (Scott Knott Test).

Seven best-performing isolates from the first experiment were selected for a second test using ^15^N labelled soil as the growth medium. All the selected isolates, except KNUST 1004, were ranked effective (with SEI > 75%) in the previous experiment. Although isolate KNUST 1030 had a SEI > 75% in the first experiment, it was not selected because re-isolation after the first experiment failed, thus the next best performing isolate from the partially effective group (i.e. KNUST 1004 with SEI = 73.6%) was selected. Significant (p < 0.05) differences were observed among isolates in terms of nodulation, shoot dry weight and nitrogen accumulated in the shoot. Nodulation in all the treatments was significantly (p < 0.05) higher than the un-inoculated control (–N). The non-nodulating (NN) soybean and common bean on the other hand did not show any signs of nodulation. The symbiotic association between three of the isolates and the test host resulted in increased nodule dry weight comparable to the control treatment inoculated with the 32H1 reference strain. Isolates that induced high nodule dry weights did not necessarily produce higher nodule numbers and vice versa (Table 3). Among the reference strains used, 32H1 was the most effective in terms of shoot dry weight while isolate KNUST 1002 produced the highest (p < 0.05) shoot dry weight among the test isolates. Considering the effect of inoculation on shoot N accumulation, isolate KNUST 1002 promoted a significant (p < 0.05) increase in N accumulation of groundnut shoot when compared to all the other test isolates (Fig. 1). The performance of isolate KNUST 1002 was not significantly different from that of the reference strain 32H1. Generally, the shoot dry weight and N accumulation of inoculated groundnut plants were superior to the reference plants (Table 3 and Fig. 1).

**Table 3:** Nodulation and shoot dry weight of inoculated groundnut and reference plants grown in ^15^N labelled soil.

Isolate/strain/reference plant	Nodule number	Nodule dry weight	Shoot dry weight
		kg ha^−1^	
KNUST 1001	341.0 e	146.6 a	2672.5 c
KNUST 1002	411.5 d	147.5 a	2867.5 b
KNUST 1003	564.8 a	132.9 a	2745.0 c
KNUST 1004	337.5 e	146.5 a	2556.3 d
KNUST 1005	456.3 c	114.8 b	2331.3 e
KNUST 1006	495.8 c	120.4 b	2687.5 c
KNUST 1007	488.3c	120.1 b	2596.3 d
NON-INOCULATED	416.0 d	87.0 c	1677.5 g
BR 10254	523.0 b	147.8 a	2340.0 e
BR 3267	499.5 c	126.4 b	2565.0 d
32H1	498.3 c	151.3 a	3146.3 a
SEMIA 6144	476.3 c	137.1 a	2193.8 f
NN common bean	–	–	466.3 i
NN Soybean	–	–	712.5 h
Sorghum	–	–	300.0 j
CV (%)	5.9	8.6	2.9

Means in the same column followed by the same letter are not significantly different at P < 0.05 (Scott Knott Test). NN: non-nodulating.

In general, the ^15^N enrichment values of inoculated plants were lower than all the reference plants. The ^15^N enrichment data showed that there were very small amounts of N derived from the soil. The level of ^15^N enrichment suggests that, for the N-rich legume seeds of NN common bean (9.3 mg N seed^−1^) and NN soybean (13.0 mg N seed^−1^), the large differences in seed N content (which were not enriched with ^15^N) were responsible for much of the isotope dilution. For this reason, only the sorghum plant (seed N content 0.62 mg N seed^−1^) was used as a reference for estimating the ^15^N enrichment of the N derived from the soil with the assumption that 50% of the shoot N was derived from the seed. The values for the percentage of nitrogen derived from the atmosphere (%Ndfa) ranged from 88 to 93% with no significant (p < 0.05) difference between treatments (Supplementary Table S2). However, there were large and significant differences between the values for the total N derived from BNF (Fig. 1). Isolate KNUST 1002 accumulated more N from BNF than any other strain except the reference strain 32H1 and most of the other strains isolated in Ghana were statistically similar to that of the reference strain BR 3267. The lowest amount of N fixed was recorded for the non-inoculated treatment.

### 3.3 Morpho – cultural and genetic characterisation of effective isolates

The seven isolates selected as most effective on groundnut grown on sterilised sand were characterised based on morpho-cultural and molecular genetic characteristics. Five isolates (KNUST 1001, 1002, 1004, 1005 and 1006) formed isolated colonies within six to seven days, alkalinized the culture medium and formed opaque-white colonies; traits that are consistent with the genus *Bradyrhizobium*. Isolates KNUST 1003 and KNUST 1007 acidified the culture medium, forming circular and elevated colonies. These fast-growing isolates, the colonies of which formed within three days, produced abundant mucus that was shiny in appearance while slow-growing isolates produced colonies with reduced mucus and were more opaque in appearance (Supplementary Table S3).

The phylogeny of the selected strains was studied by analysing the near-complete sequence of their 16S rRNA gene and ITS region. Basic Local Alignment Search Tool (BLAST) analysis of the 16S rRNA sequences confirmed that isolate KNUST 1001, 1002, 1004, 1005 and 1006 belonged to the genus *Bradyrhizobium*. The fast-growing isolates KNUST 1003 and 1007 were highly similar to members of the genus *Rhizobium* (Supplementary Fig. S3a and b).

**Fig. 2:**
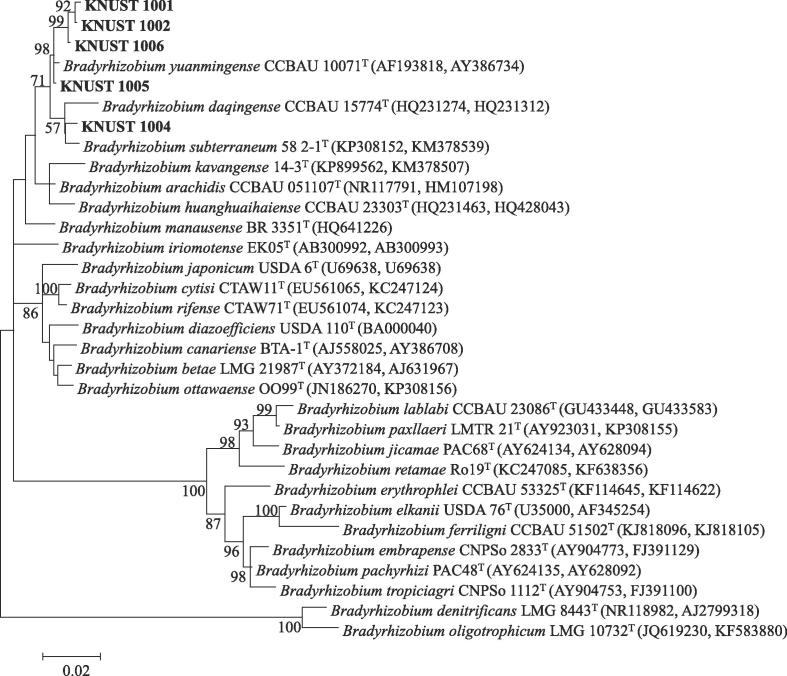
Unrooted maximum likelihood phylogenetic tree based on concatenated 16S rRNA gene and ITS sequences showing relationships among isolates and type-strains ^(T)^ of the genus *Bradyrhizobium*. Bootstrap values were inferred from 500 replicates and are indicated at the tree nodes when ≥50%. GenBank accession numbers are provided in parentheses. The bar represents two estimated substitutions per 100 nucleotide positions.

Within the genus *Bradyrhizobium,* 16S rRNA sequences are too conserved to permit for a more detailed phylogenetic classification at the species level. The ITS sequence between the 16S and 23S rRNA genes can be used to improve this phylogenetic resolution. Therefore, in this study, phylogenetic analysis was performed on the concatenated sequences of the 16S rRNA gene (1243 nt) and the ITS region (1027 nt) giving a total of 2270 nt. In this analysis, the five *Bradyrhizobium* isolates from this study clustered together on the same branch with close relation to *B. yuanmingense* CCBAU 10071^T^, *Bradyrhizobium daqingense* CCBAU 15774^T^ and *Bradyrhizobium subterraneum* 48 2-1^T^ with bootstrap support of at least 71% and with nucleotide sequence similarity values between 99.1 and 99.3% (Fig. 2, Supplementary Table S4). Generally, the concatenated analyses of 16S rRNA gene and ITS region revealed a clearer relationship between clustering of *Bradyrhizobium* strains or isolates compared to their individual analyses (Supplementary Fig. S3a and S4).

### 3.4 Analyses of symbiotic genes

**Fig. 3:**
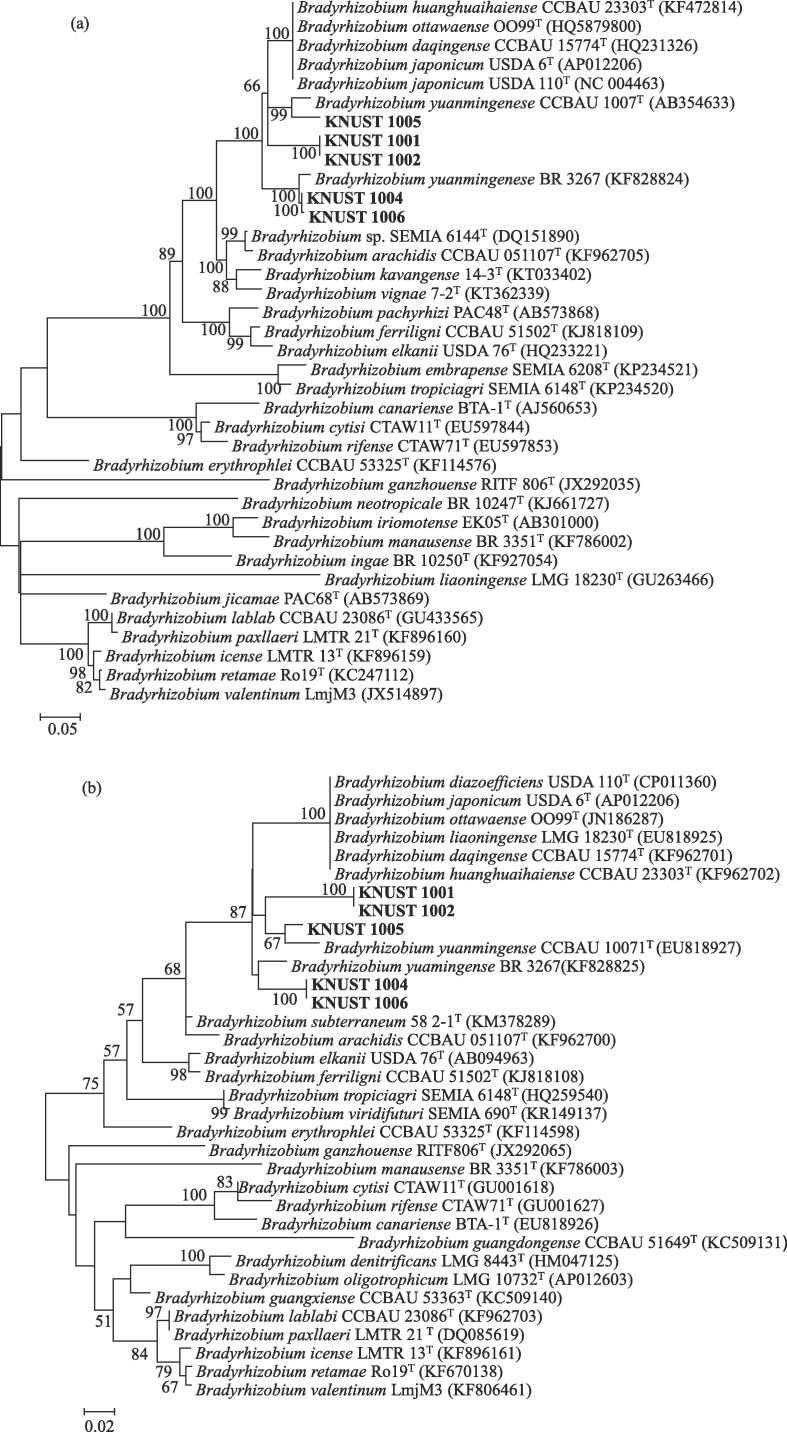
Unrooted maximum likelihood phylogenetic tree based on *nodC* (a) and *nif*H (b) genes showing relationships among isolates and type-strains (^T^) of the genus *Bradyrhizobium*. Bootstrap values were inferred from 500 replicates and are indicated at the tree nodes when ≥50%. GenBank accession numbers are provided in the parenthesis. The bar represents five or two estimated substitutions per 100 nucleotide positions.

The phylogenetic relationship of the *nodC* gene from the novel *Bradyrhizobium* isolates in relation to validly described species was studied. The *nodC* phylogenetic analyses placed two of the isolates, KNUST 1004 and 1006, on a branch together with *B. yuanmingense* BR 3267^T^ with 100% bootstrap support (Fig. 3a). The isolates KNUST 1001, 1002 and 1005 were together on a branch with their sequences most closely related to *B. yuanmingense* CCBAU 1007^T^, *Bradyrhizobium ottawaense* OO99^T^, *Bradyrhizobium japonicum* USDA 6^T^, *Bradyrhizobium huanghuaihaiense* CCBAU 23303^T^ and *Bradyrhizobium denitrificans* LMG 8443^T^, with 65% bootstrap support. In agreement with the *nodC* phylogeny, phylogenetic analyses of the *nifH* gene also placed the isolate KNUST 1005 in a branch together with *B. yuanmingense* CCBAU 10071^T^ followed by the inclusion of isolates KNUST 1001 and 1002. Isolates KNUST 1004 and 1006 also shared close relation to *B. yuanmingense* BR 3267^T^ (Fig. 3b). Therefore, in general, the *nodC* and *nifH* phylogenies were congruent with the phylogeny estimated based on the concatenated 16S rRNA and ITS sequences.

## 4 Discussion

### 4.1 Authentication and symbiotic potential of isolates

In tropical soils, there is an enormous diversity of rhizobia with different nodulation capacities, which forms a natural reserve of germplasm for the selection of strains with desired characteristics (Dilworth et al., 2001). When assessing the relationship between rhizobia and their host, infectivity and symbiotic effectiveness are the two essential features commonly considered (Brockwell, 1998). The symbioses between legumes and rhizobium must be effective for enhanced BNF and subsequent yield improvement to be realized. In this study, a preliminary screening for authentic rhizobia was performed using cowpea in growth pouches because this species is easier to grow under such conditions than the target species groundnut. The variation in numbers of infective isolates between cowpea and groundnut reflects differences in host-range among bacteria and host plant species and also confirms earlier studies that report that groundnut has a more restrictive nodulation pattern than cowpea (Thies et al., 1991). Nevertheless, the inoculation experiments described herein demonstrate that it was possible to obtain several effective rhizobia for groundnut inoculation, notably *Bradyrhizobium* sp. KNUST 1002, which caused increments in nodulation, shoot dry weight and amount of N fixed in both experiments. The increase in nodulation observed following inoculation may be attributed to the favorable chemical properties of the study soil (such as pH). The possibility of achieving good nodulation and N_2_ fixation above a pH of 5.2 has been reported by Hungria and Vargas (2000) and which was the case in this study. The outstanding performance of *Bradyrhizobium* sp. KNUST 1002 could imply that it was more compatible with the legume host than the other test isolates.

The effectiveness of the symbiotic association between the infective isolates and their host in this study revealed varying effectiveness classes with some test isolates resulting in significantly higher SEI than the reference strains used. Useful variations in characteristics required in inoculant strains such as symbiotic effectiveness have also been observed within the natural pool of soil rhizobia (O’Hara et al., 2002). Nitrogen fixation efficiency has been found to be diverse, ranging from symbiotic interactions leading to little or no nitrogen fixation, to those that obtain nitrogen in levels equivalent to or even greater than plants treated with mineral N (Terpolilli et al., 2008). Similar results have been reported in other studies carried out to evaluate rhizobium cultures of various tropical legumes for their symbiotic capacity (Florentino et al., 2010, Marra et al., 2012). Additionally, the uneven distribution of effective isolates demonstrated in the principal component analysis (Supplementary Fig. S2) highlights a wide variation in terms of geographic distribution and symbiotic performance (Abaidoo et al., 2007). In order to overcome sub-optimal N_2_ fixation, the need arises to acquire rhizobium with high N_2_ fixing ability that are also well adapted to the prevailing environment (Yates et al., 2016). Estimation of the contribution of strains to nitrogen fixation has been based on methods such as N_2_ balance, N_2_ difference, ^15^N natural abundance, ureide analyses, acetylene reductase assay, hydrogen evolution and ^15^N isotope dilution method (Unkovich et al., 2008). The latter method was employed in this study to quantify the amount of N_2_ fixed for the selected effective isolates (experiment 2). Three reference plants were included in this experiment to estimate % Ndfa, because of the difficulty in directly determining which reference crop would accumulate N with the same ^15^N enrichment as the legume crop. Boddey et al. (1995) thus recommended that several reference plants should be utilized to produce individual estimates of BNF contribution with the range of these estimates considered as an index of their accuracy. The lower ^15^N enrichment recorded by inoculated plants implies that they received contributions of unlabeled N through BNF. On the other hand, the estimates of ^15^N enrichments of the NN legume reference plants were extremely high and which could be due to their large seed N contents. Naegle et al. (2005), indicated that the response of seedlings’ (of soybean) to available N is strongly related to available seed N resources. Since the seed N content of the NN reference plants were so high, and the availability of labelled soil N was so low, the results suggest that the N derived from the seed was considerably higher than 50%. The estimated ^15^N enrichments for these two NN legume crops of approximately 0.19–0.20 atom% excess were far higher than that of the small-seeded sorghum and as such, it is thought impossible that these NN legumes obtained only half of their N from seed reserves. Therefore, to avoid over estimation of BNF, the proportion of N derived from the atmosphere was estimated using sorghum as the reference crop.

The insignificant differences observed for the various treatments in terms of the ^15^N enrichment was not surprising since all the isolates selected for this experiment were potentially effective. The un-inoculated treatment resulted in the proportion of N derived from the atmosphere that was similar to all other treatments, but the amount of BNF contributed by the former was significantly lower. The results also show that, the proportion of plant accumulated N derived from BNF by inoculated plants ranged between 88 and 93%. The large differences between the different strains was apparent only in the total accumulated N and the total N derived from BNF. The *Bradyrhizobium* strain 32H1 was the best and most consistent reference strain in all parameters measured supporting the claim that it is an effective strain on groundnut (Urtz and Elkan, 1996). All isolates except KNUST 1005 performed similar to the reference strain BR 3267. The results from this study indicate the presence of indigenous rhizobia strains with highly effective symbiotic capacities that can be used as inoculants.

### 4.2 Genetic characterisation of effective isolates

Morpho-cultural characterisation of isolates used in this study indicated that they belong to the genera *Bradyrhizobium* and *Rhizobium* and this was confirmed by BLAST analysis of 16S rRNA gene sequences. Groundnut has been found previously to form associations with strains from the *Rhizobium* genus in addition to *Bradyrhizobium* symbionts (Van Rossum et al., 1995, Urtz and Elkan, 1996, Yang et al., 2005). Five out of seven isolates in this study belong to the *Bradyrhizobium* genus affirming the observation by several authors that bacteria associated with peanut are predominantly *Bradyrhizobium* (Van Rossum et al., 1995, Zhang et al., 1999, Yang et al., 2005). The classification of novel species has been based on a polyphasic approach, which considers phenotypic and genetic characteristics (Vandamme et al., 1996). This approach employs the sequencing of 16S rRNA gene as the backbone of genetic classification (Garrity and Holt, 2001). However, this gene has been found to be limited in delineating the diversity within *Bradyrhizobium* at the species level (Wang and Martínez-Romero, 2000) corroborating the findings in this study where diversity within *Bradyrhizobium* isolates was not clearly defined. The *Rhizobium* isolates characterised in this study showed close relation to the *Rhizobium tropici* group (Dall’Agnol et al., 2013). Strains belonging to this group are characterised with broad-host-range, high tolerance to environmental stress and genetic stability (Hungria et al., 2000, Hungria et al., 2003). This observation is interesting since the isolates in this study were obtained from areas with harsh environmental conditions. The analysis of concatenated 16S rRNA and ITS regions in this study revealed that the isolates shared more than 95.5% similarity to the *B. yuanmingense* strain CCBAU 1007^T^; implying that these isolates belong to this species (Willems et al., 2001). Willems et al. (2003) reported that the ITS gene sequencing and DNA-DNA hybridization shared a high correlation such that a sequence similarity of more than 95.5% shared by strains indicates that they belong to the same genospecies and have more than 60% DNA-DNA hybridization.

Symbiotic genes, on the other hand, have been found useful in the determination of host range, nodulation capacity and symbiovars between rhizobia and legumes (Rogel et al., 2011). The observation from the analyses of symbiotic genes of isolates in this study revealed that some areas in northern Ghana harbour strains related to *B. yuanmingense* in addition to the already identified geographical origins of this species (So et al., 1994, Vinuesa et al., 2005, Ormeno-Orrillo et al., 2006, Gu et al., 2007, Steenkamp et al., 2008, Leite et al., 2017). This suggests that strains of this species may be widely distributed in nature. Although *nifH* phylogeny is characterised by lateral gene transfer related to the host (Vinuesa et al., 2005), isolates that effectively nodulated groundnut in this study did not necessarily show any close relation with typical groundnut symbionts such as *Bradyrhizobium arachidis* (Wang et al., 2013), *Bradyrhizobium subterraneum* (Grönemeyer et al., 2015) and *Bradyrhizobium vignae* (Grönemeyer et al., 2016). This observation maybe because effective isolates used in this study were originally obtained from nodules of cowpea plants.

To this end, *B. yuanmingense* has been confirmed to be an important micro-symbiont of groundnut as reported previously in other studies (Gu et al., 2007, Steenkamp et al., 2008, Leite et al., 2017).

## 5 Conclusion

Increased nodulation, biomass production and N accumulation in soil-grown groundnut were achieved after inoculation with native rhizobium strains of northern Ghana. Among the isolates tested, KNUST 1002 was highly effective, performing similar to the groundnut reference strain 32H1. Apart from two *Rhizobium* isolates (KNUST 1003 and 1007), all the strains selected in this study were closely related to *B. yuanmingense*, confirming this species as a major micro-symbiont of groundnut.
