# Supplementary material for: Bacteria related to Bradyrhizobium yuanmingense from Ghana are effective groundnut micro-symbionts
Source: Appl Soil Ecol. 2018 Jun;127:41–50. doi: 10.1016/j.apsoil.2018.03.003 (PMC5989812; doi:10.1016/j.apsoil.2018.03.003)
Supplement: Supplementary data 1 [file mmc1.pdf]

Applied Soil Ecology

SUPPLEMENTARY MATERIAL

Title: Bacteria related to *Bradyrhizobium yuanmingense* from Ghana are effective groundnut micro-symbionts

Authors: Ophelia Osei, Robert C. Abaidoo, Benjamin. D. K. Ahiabor, Robert M. Boddey and Luc F. M. Rouws

Table S1

Principal component analysis explaining the variation between variables

	Eigen Value	Percent of Variance	Cumulative Percentage
1	3.36	**84.1**	84.05
2	0.42	**10.5**	**94.5**
3	0.20	5.052	99.6
4	0.016	0.421	100.0

Table S2

^{15}N enrichment and proportion of nitrogen derived from air (%Ndfa) of groundnut and reference plant

Isolate/strain/reference plant	Atom % ^{15}N excess	% Ndfa calculated using sorghum as the non-N_2- fixing reference plant
KNUST 1001	0.00592 c	91.8 a
KNUST 1002	0.00872 c	87.8 a
KNUST 1003	0.00613 c	91.4 a
KNUST 1004	0.00550 c	92.3 a
KNUST 1005	0.00664 c	90.8 a
KNUST 1006	0.00551 c	92.3 a
KNUST 1007	0.00661 c	90.7 a

Non-inoculated	0.00692 c	90.2 a
BR 10254	0.00594 c	91.7 a
BR 3267	0.00536 c	92.5 a
32H1	0.00601 c	91.5 a
SEMIA 6144	0.00542 c	92.4 a
Sorghum	0.07125 b	-
NN Soybean	0.18750 a	-
NN Common bean	0.20574 a	-
CV (%)	48.8	

Means in the same column followed by the same letter are not significantly different at P< 0.05 (Scott Knott Test).

Table S3

Morpho-cultural characteristics of effective isolates

Isolates	Days to single colony(C) appearance	pH reaction	Colony morphology	Colony transparency/Colour	Mucus produced
KNUST 1001	6	Alkaline	Circular	Opaque/ White	Less
KNUST 1002	7	Alkaline	Circular	Opaque/ White	Less
KNUST 1003	3	Acidic	Circular	Translucent/ Yellow	More
KNUST 1004	7	Alkaline	Irregular	Opaque/ White	Less
KNUST 1005	7	Alkaline	Circular	Opaque/ White	Less
KNUST 1006	7	Alkaline	Circular	Opaque/ White	Less
KNUST 1007	3	Acidic	Circular	Translucent/ Yellow	More

Table S4

Percentage nucleotide similarity at different genetic loci between already described *Bradyrhizobium* strains and isolates from this study. The last column shows values for concatenated sequences of two loci. Similarity values were determined using the Mega 7 software. -: data not available

Strains/ genes	**16S rRNA**	**ITS**	***nodC***	***nifH***	**16S rRNA, ITS**
Bradyrhizobium yuanmingense CCBAU 10071 ^{T}					
KNUST 1001	99.75%	97.52%	89.74%	92.54%	99.05%
KNUST 1002	99.59%	97.88%	89.74%	92.54%	99.16%
KNUST 1004	99.59%	97.89%	90.28%	92.19%	99.16%
KNUST 1005	99.75%	99.83%	94.33%	96.62%	99.78%
KNUST 1006	99.43%	98.59%	90.03%	92.19%	99.33%
Bradyrhizobium yuanmingense BR3267	99.26%	-	89.25%	93.12%	-
Bradyrhizobium japonicum SEMIA 6144 ^{T}	98.60%	-	86.29%	-	-
Bradyrhizobium sp. BR 10254 ^{T}	98.68%	-	-	-	-
Bradyrhizobium arachidis CCBAU 051107 ^{T}	98.85%	98.24%	85.43%	91.88%	98.66%
Bradyrhizobium viridifuturi SEMIA 690 ^{T}	96.99%	-	-	88.53%	-
Bradyrhizobium embrapense SEMIA 6208 ^{T}	96.99%	86.61%	78.34%	-	94.15%
Bradyrhizobium subterraneum 58 2-1 ^{T}	99.51%	97.17%	-	93.09%	98.82%

Bradyrhizobium betae LMG 21987 T	99.01%	94.58%	-	-	97.58%
Bradyrhizobium canariense BTA-1 T	99.10%	95.69%	65.71%	83.85%	97.97%
Bradyrhizobium cytisi CTAW11 T	99.01%	94.94%	68.72%	83.32%	97.75%
Bradyrhizobium daqingense CCBAU 15774 T	99.43%	96.07%	91.57%	91.40%	98.32%
Bradyrhizobium denitrificans LMG 8443 T	98.18%	78.59%	-	85.01%	92.44%
Bradyrhizobium elkanii USDA 76 T	96.73%	88.28%	80.49%	89.17%	94.45%
Bradyrhizobium erythrophlei CCBAU 53325 T	96.99%	89.04%	71.20%	88.04%	94.87%
Bradyrhizobium ferriligni CCBAU 51502 T	94.89%	86.82%	81.03%	89.63%	92.67%
Bradyrhizobium ganzhouense RITF806 T	99.18%	-	63.98%	85.38%	-
Bradyrhizobium guangdongense CCBAU 51649 T	99.26%	-	-	82.43%	-
Bradyrhizobium guangxiense CCBAU 53363 T	99.18%	-	-	85.58%	-
Bradyrhizobium huanghuaihaiense CCBAU 23303 T	98.68%	97.17%	91.57%	91.40%	98.15%
Bradyrhizobium icense LMTR 13 T	96.90%	-	68.99%	85.71%	-
Bradyrhizobium ingae BR 10250 T	98.35%	-	63.82%	-	-
Bradyrhizobium iriomotense EK05 T	98.35%	95.51%	63.34%	-	97.46%
Bradyrhizobium japonicum USDA 6 T	99.43%	95.34%	91.57%	91.40%	98.09%
Bradyrhizobium jicamae PAC 68 T	96.65%	88.04%	64.54%	-	94.33%
Bradyrhizobium kavangense 14-3 T	98.93%	97.34%	85.72%	-	98.43%

Bradyrhizobium lablabi CCBAU 23086 [T]	96.82%	87.25%	69.66%	85.73%	94.21%
Bradyrhizobium liaoningense LMG 18230 [T]	99.51%	95.51%	59.52%	91.40%	-
Bradyrhizobium manausense BR 3351 [T]	99.18%	96.25%	63.77%	81.61%	98.26%
Bradyrhizobium oligotrophicum LMG 10732 [T]	98.43%	76.86%	-	84.87%	92.25%
Bradyrhizobium ottawaense OO 99 [T]	99.34%	95.69%	91.57%	91.40%	98.15%
Bradyrhizobium pachyrhizi PAC 48 [T]	96.82%	87.43%	81.35%	-	94.27%
Bradyrhizobium paxllaeri LMTR 21 [T]	96.90%	87.01%	69.63%	85.73%	94.21%
Bradyrhizobium retamae Ro 19 [T]	97.24%	86.76%	68.56%	86.14%	94.38%
Bradyrhizobium rifense CTAW 71 [T]	99.10%	94.74%	67.53%	83.38%	97.69%
Bradyrhizobium valentinum LmjM3	96.82%	-	68.85%	86.21%	-
Bradyrhizobium vignae 7-2 [T]	99.01%	97.88%	85.70%	-	-
Bradyrhizobium tropiciagri SEMIA 6148 [T]	96.21%	87.38%	80.58%	88.08%	93.84%
Bradyrhizobium diazoefficiens USDA 110 [T]	99.01%	94.20%	91.57%	91.40%	97.52%
Bradyrhizobium neotropicale BR 10247 [T]	96.39%	95.34%	62.87%	-	-
Microvirga vignae BR 3299[T]	90.47%	-	-	-	-

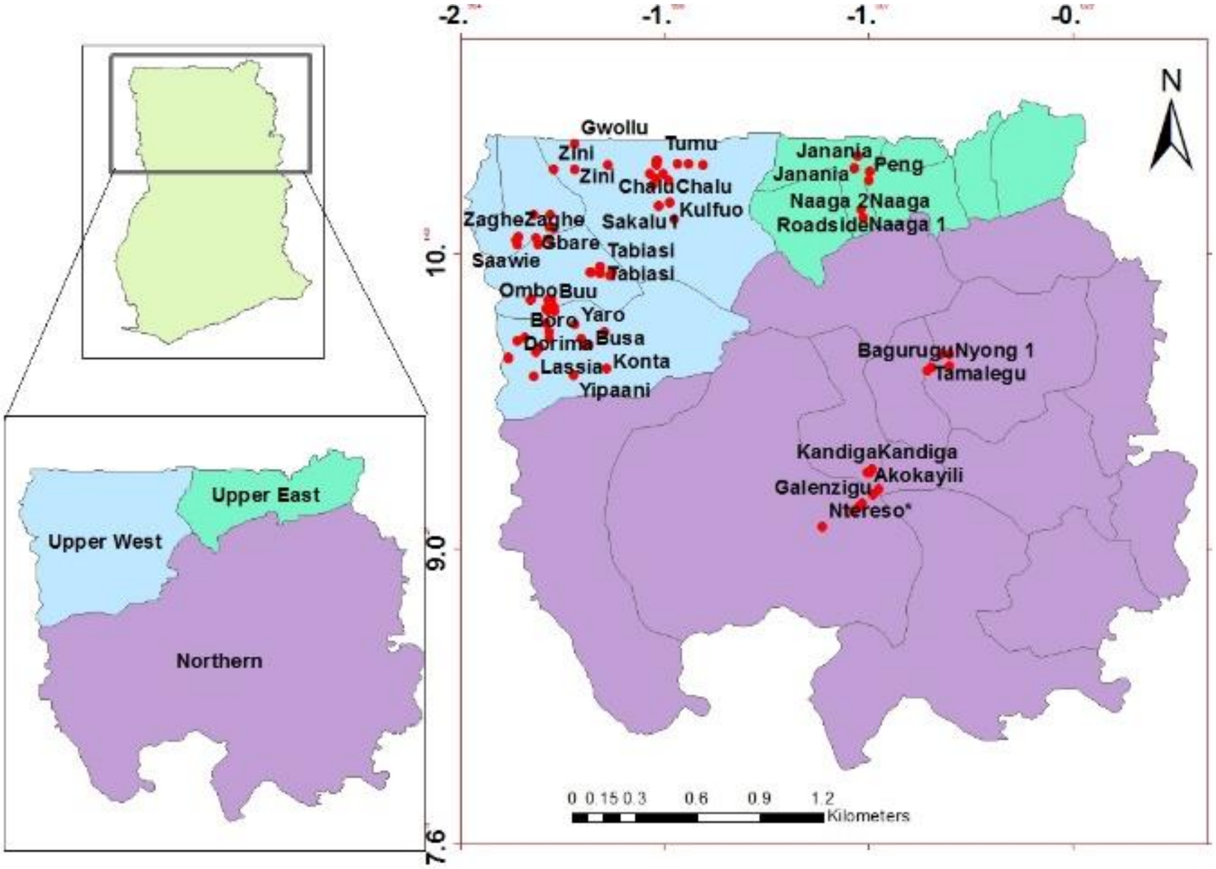

Fig. S1. Locations of cowpea and groundnut nodule sampling sites in northern Ghana representing the major legume growing areas.

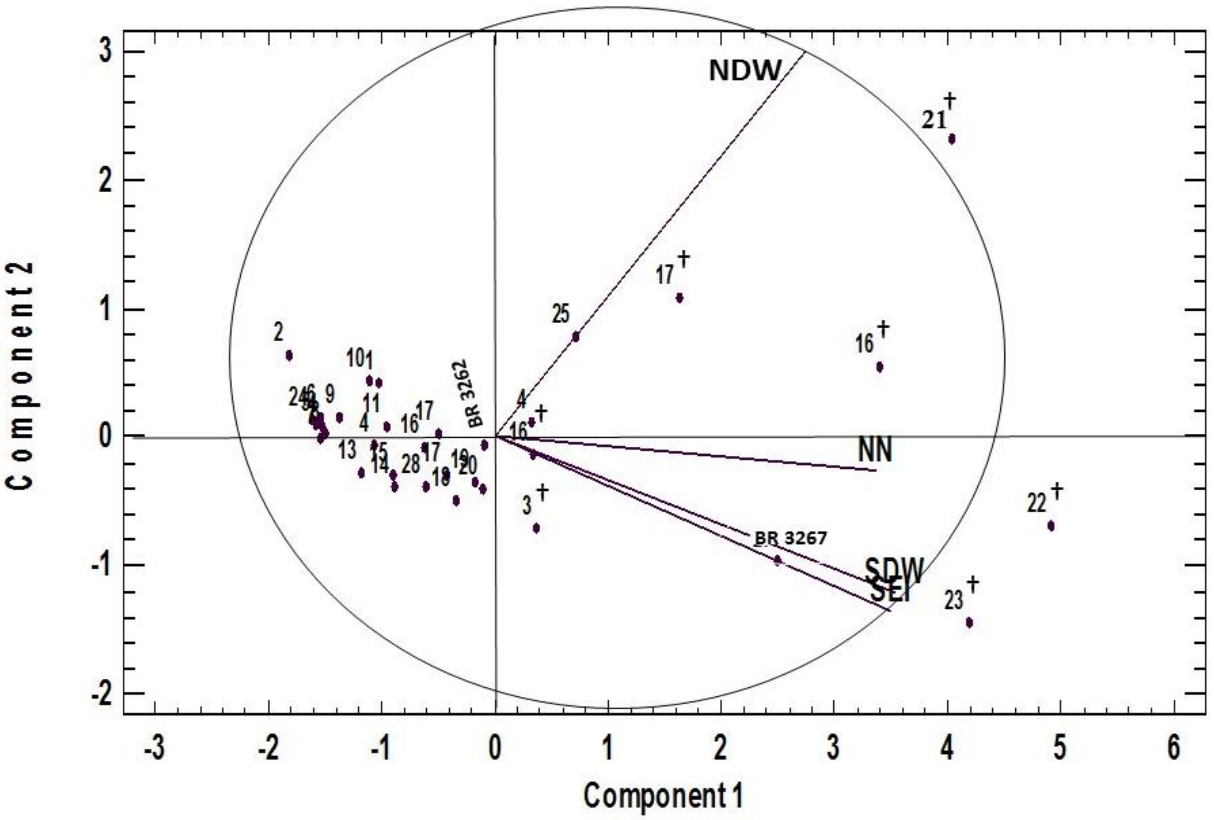

Fig. S2. Principal component analysis for the relationships among sampling sites as sources of effective or ineffective isolates. Reference strains *Bradyrhizobium yuanmingense* strain BR 3267 and *Bradyrhizobium pachyrhizi* strain BR 3262 represent sources of good perfoming strains. Numbers represent sampling sites: 1- Tamale; 2- Gbare; 3- Akokayilli; 4- Kandiga; 5- Naaga; 6- Pishegu; 7- Chiatanga; 8- Punyoro; 9- Dorima; 10- Varimpere; 11- Tabiasi; 12- Kandiga; 13- Tabiasi4 14- Varimpere 2; 15- Kuncheni; 16- Kandiga 2; 17- Boro; 18- Zaguo daryiri; 19- Zaguo daryiri; 20- Saawie; 21- Punyoro kb; 22- Konta; 23- Yipaani; 24- Tabiasi 2; 25- Kpalga.

† Isolates selected for second experiment. SDW: shoot dry weight, NN: nodule number, NDW: nodule dry weight, SEI: symbiotic effectiveness index

Fig S3a.

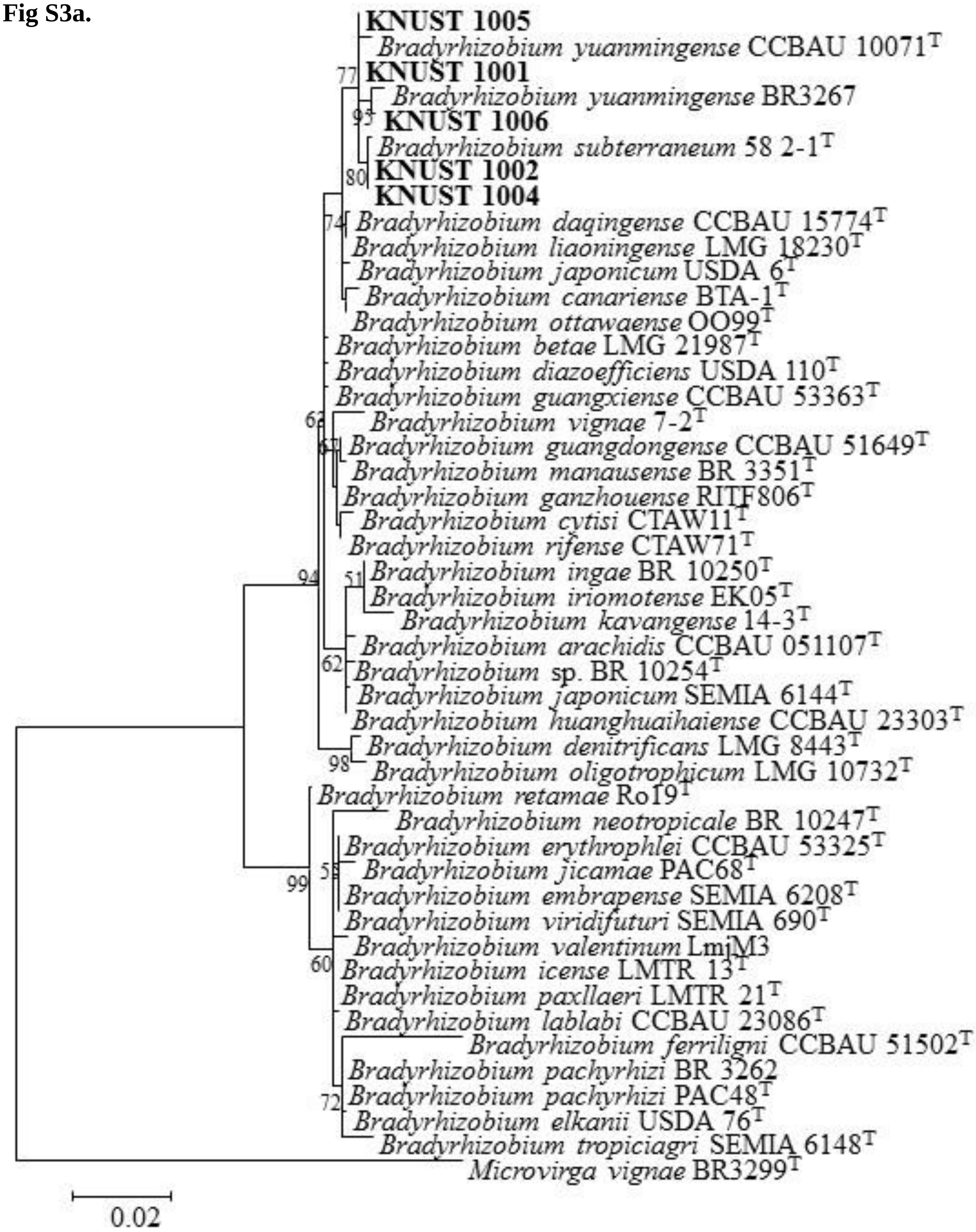
KNUST 1005
Bradyrhizobium yuanmingense CCBAU 10071T
77
KNUST 1001
Bradyrhizobium yuanmingense BR3267
95
KNUST 1006
Bradyrhizobium subterraneum 58 2-1T
80
KNUST 1002
KNUST 1004
74
Bradyrhizobium daqingense CCBAU 15774T
Bradyrhizobium liaoningense LMG 18230T
Bradyrhizobium japonicum USDA 6T
Bradyrhizobium canariense BTA-1T
Bradyrhizobium ottawaense OO99T
Bradyrhizobium betae LMG 21987T
Bradyrhizobium diazoefficiens USDA 110T
Bradyrhizobium guangxiense CCBAU 53363T
62
Bradyrhizobium vignae 7-2T
61
Bradyrhizobium guangdongense CCBAU 51649T
Bradyrhizobium manausense BR 3351T
Bradyrhizobium ganzhouense RITF806T
Bradyrhizobium cytisi CTAW11T
Bradyrhizobium rifense CTAW71T
94
51
Bradyrhizobium ingae BR 10250T
Bradyrhizobium iriomotense EK05T
Bradyrhizobium kavangense 14-3T
Bradyrhizobium arachidis CCBAU 051107T
62
Bradyrhizobium sp. BR 10254T
Bradyrhizobium japonicum SEMIA 6144T
Bradyrhizobium huanghuaihaiense CCBAU 23303T
Bradyrhizobium denitrificans LMG 8443T
98
Bradyrhizobium oligotrophicum LMG 10732T
Bradyrhizobium retamae Ro19T
Bradyrhizobium neotropicale BR 10247T
Bradyrhizobium erythrophlei CCBAU 53325T
58
99
Bradyrhizobium jicamae PAC68T
Bradyrhizobium embrapense SEMIA 6208T
Bradyrhizobium viridifuturi SEMIA 690T
Bradyrhizobium valentinum LmjM3
60
Bradyrhizobium icense LMTR 13T
Bradyrhizobium paxllaeri LMTR 21T
Bradyrhizobium lablabi CCBAU 23086T
Bradyrhizobium ferriligni CCBAU 51502T
Bradyrhizobium pachyrhizi BR 3262
72
Bradyrhizobium pachyrhizi PAC48T
Bradyrhizobium elkanii USDA 76T
Bradyrhizobium tropiciagri SEMIA 6148T
Microvirga vignae BR3299T
0.02

Fig S3b.

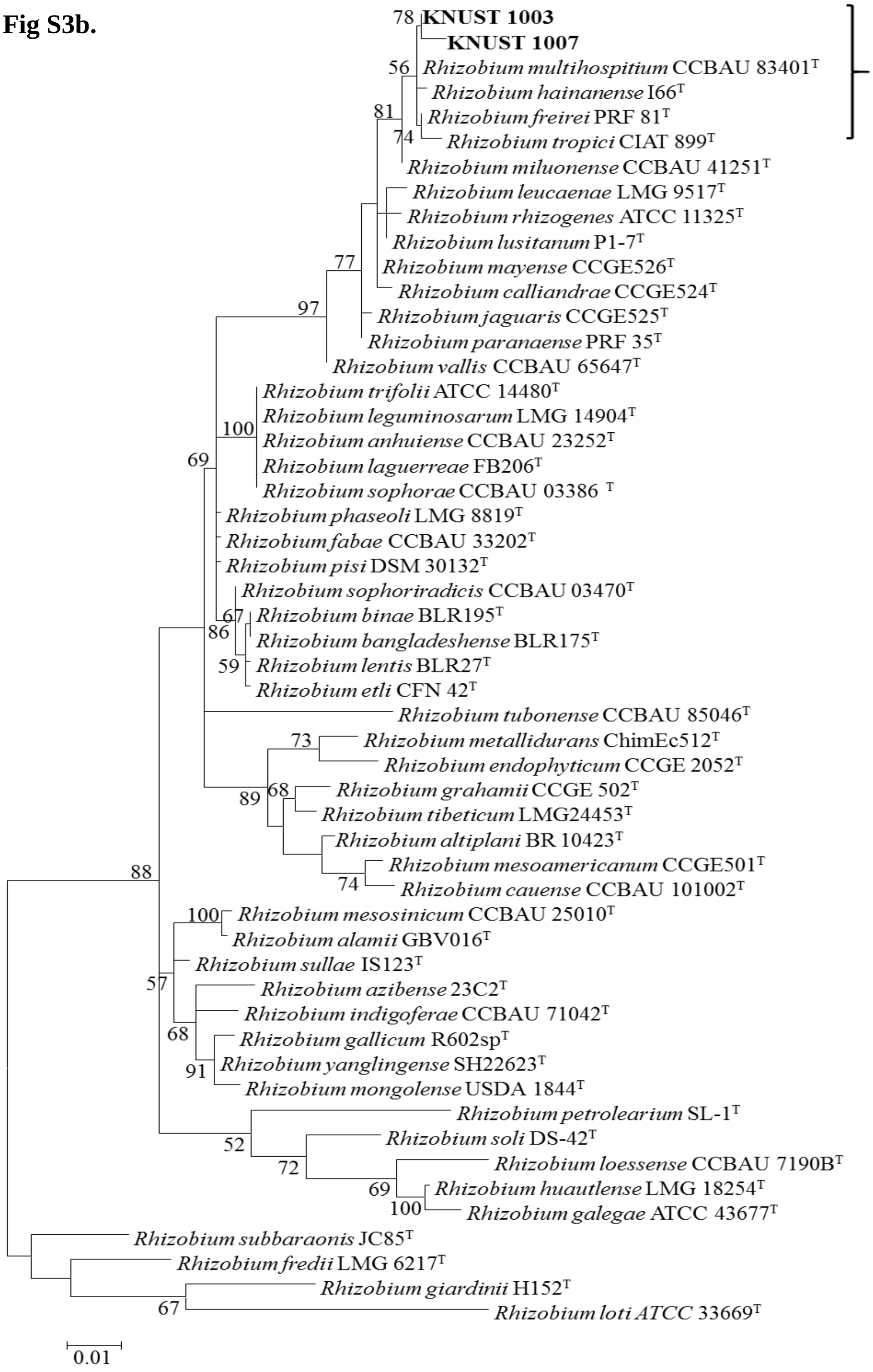
78
KNUST 1003
KNUST 1007
56
Rhizobium multihospitium CCBAU 83401T
Rhizobium hainanense I66T
81
Rhizobium freirei PRF 81T
74
Rhizobium tropici CIAT 899T
Rhizobium miluonense CCBAU 41251T
Rhizobium leucaenae LMG 9517T
Rhizobium rhizogenes ATCC 11325T
Rhizobium lusitanum P1-7T
77
Rhizobium mayense CCGE526T
Rhizobium calliandrae CCGE524T
97
Rhizobium jaguaris CCGE525T
Rhizobium paranaense PRF 35T
Rhizobium vallis CCBAU 65647T
Rhizobium trifolii ATCC 14480T
Rhizobium leguminosarum LMG 14904T
100
Rhizobium anhuiense CCBAU 23252T
69
Rhizobium laguerreae FB206T
Rhizobium sophorae CCBAU 03386 T
Rhizobium phaseoli LMG 8819T
Rhizobium fabae CCBAU 33202T
Rhizobium pisi DSM 30132T
Rhizobium sophoriradicis CCBAU 03470T
67
Rhizobium binae BLR195T
86
Rhizobium bangladeshense BLR175T
59
Rhizobium lentis BLR27T
Rhizobium etli CFN 42T
Rhizobium tubonense CCBAU 85046T
73
Rhizobium metallidurans ChimEc512T
Rhizobium endophyticum CCGE 2052T
68
Rhizobium grahamii CCGE 502T
89
Rhizobium tibeticum LMG24453T
Rhizobium altiplani BR 10423T
Rhizobium mesoamericanum CCGE501T
88
74
Rhizobium cauense CCBAU 101002T
100
Rhizobium mesosinicum CCBAU 25010T
Rhizobium alamii GBV016T
Rhizobium sullae IS123T
57
Rhizobium azibense 23C2T
Rhizobium indigoferae CCBAU 71042T
68
Rhizobium gallicum R602spT
Rhizobium yanglingense SH22623T
91
Rhizobium mongolense USDA 1844T
Rhizobium petrolearium SL-1T
52
Rhizobium soli DS-42T
72
Rhizobium loessense CCBAU 7190BT
69
Rhizobium huautlense LMG 18254T
100
Rhizobium galegae ATCC 43677T
Rhizobium subbaraonis JC85T
Rhizobium fredii LMG 6217T
Rhizobium giardinii H152T
67
Rhizobium loti ATCC 33669T
0.01

Fig. S3. Maximum likelihood phylogenetic tree based on 16S rRNA gene showing relationships between isolates, type species (^{T}) and reference strains of the genera *Bradyrhizobium* (a) and *Rhizobium* (b). The phylogenetic tree was built based on the Tamura 3-parameter model. Bootstrap values were inferred from 500 replicates and are indicated at the tree nodes when ≥50%. The bar represents two or one estimated substitutions per 100 nucleotide positions.

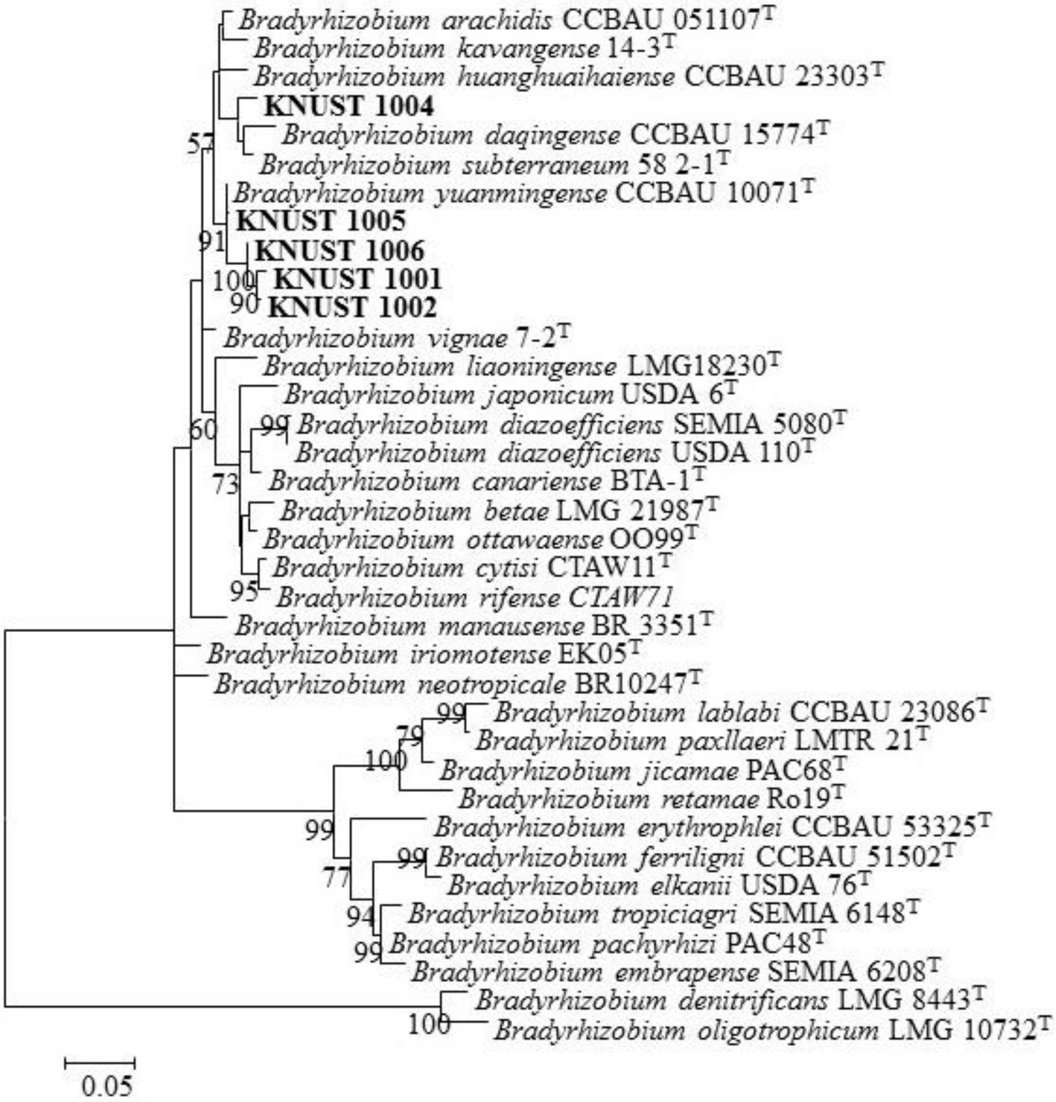

Fig. S4. Maximum likelihood phylogenetic tree based on ITS region showing relationships between isolates, type species (^{T}) and reference strains of the genus *Bradyrhizobium*. The phylogenetic tree was built based on the Kimura 2-parameter model. Bootstrap values were inferred from 500 replicates and are indicated at the tree nodes when ≥50%. The bar represents five or two estimated substitutions per 100 nucleotide positions.
